# Blood Biomarkers and Metabolomic Profiling for the Early Diagnosis of Vancomycin-Associated Acute Kidney Injury: A Systematic Review and Meta-Analysis of Experimental Studies

**DOI:** 10.3390/jpm12091397

**Published:** 2022-08-28

**Authors:** Eleni Laou, Theodoros Mavridis, Nikolaos Papagiannakis, Gwendolyn Pais, Alberto Chighine, Jack Chang, Emanuela Locci, Ernesto D’Aloja, Marc Scheetz, Athanasios Chalkias, Theodoros Xanthos

**Affiliations:** 1 Department of Anesthesiology, Faculty of Medicine, University of Thessaly, 41500 Larisa, Greece; 2 First Department of Neurology, Eginition University Hospital, Medical School, National and Kapodistrian University of Athens, 15772 Athens, Greece; 3 Department of Pharmacy Practice, Pharmacometric Center of Excellence, Midwestern University Chicago College of Pharmacy, Downers Grove, IL 60515, USA; 4 Department of Medical Sciences and Public Health, Section of Legal Medicine, University of Cagliari, 09124 Cagliari, Italy; 5 Northwestern Memorial Hospital, Chicago, IL 60611, USA; 6 Outcomes Research Consortium, Cleveland, OH 44195, USA; 7 School of Health sciences, University of West Attica, 12243 Athens, Greece

**Keywords:** acute kidney injury, vancomycin, animal models, translational research, blood biomarkers

## Abstract

Background: several blood-based biomarkers have been proposed for predicting vancomycin-associated kidney injury (VIKI). However, no systematic analysis has compared their prognostic value. Objective: this systematic review and meta-analysis was designed to investigate the role of blood biomarkers and metabolomic profiling as diagnostic and prognostic predictors in pre-clinical studies of VIKI. Methods: a systematic search of PubMed was conducted for relevant articles from January 2000 to May 2022. Animal studies that administered vancomycin and studied VIKI were eligible for inclusion. Clinical studies, reviews, and non-English literature were excluded. The primary outcome was to investigate the relationship between the extent of VIKI as measured by blood biomarkers and metabolomic profiling. Risk of bias was assessed with the CAMARADES checklist the SYRCLE’s risk of bias tool. Standard meta-analysis methods (random-effects models) were used. Results: there were four studies for the same species, dosage, duration of vancomycin administration and measurement only for serum creatine and blood urea nitrogen in rats. A statistically significant increase was observed between serum creatinine in the vancomycin group compared to controls (pooled *p* = 0.037; Standardized Mean Difference: 2.93; 95% CI: 0.17 to 5.69; I^2^ = 92.11%). Serum BUN levels were not significantly different between control and vancomycin groups (pooled *p* = 0.11; SMD: 3.05; 95% CI: 0.69 to 6.8; I^2^ = 94.84%). We did not identify experimental studies using metabolomic analyses in animals with VIKI. Conclusions: a total of four studies in rodents only described outcomes of kidney injury as defined by blood biomarkers. Blood biomarkers represented included serum creatinine and BUN. Novel blood biomarkers have not been explored.

## 1. Introduction

Acute kidney injury (AKI) occurs in 13–78% of critically ill patients [1]. Antibiotics are a common trigger but modifiable risk factor for AKI and several randomized clinical trials have found that vancomycin-associated kidney injury (VIKI) is more common than kidney injury caused by other agents [2,3,4]. Indeed, VIKI is a clinically relevant entity with rates varying from 5% to 43%, often develops within the first 5 days of therapy, and may result in chronic renal failure, prolonged hospital stays, and increased mortality [3,4,5,6,7,8,9]. 

Vancomycin has demonstrated histopathological damage in humans and animals, and the incidence of VIKI increases with higher vancomycin concentrations and doses [10,11,12,13,14]. Specifically, several animal and in vivo studies have been designed to investigate the nephrotoxic effects of vancomycin. Some suggest oxidative stress, increased lipid peroxidation and expression of inflammatory cytokines, regulation of ROS/NF-κB pathway, and proximal renal tubular cell necrosis by vancomycin accumulation as mechanisms of nephrotoxicity [15,16]. Other studies have shown that the suppression of autophagy and altered gene expression mediate renal tubular cell apoptosis [17]. These experimental studies have strengthened the evidence revealing the relationship between the exposure of vancomycin in the kidney and toxicity of vancomycin at clinically relevant concentrations [18,19,20].

The pathophysiology of VIKI is not entirely understood, but mounting evidence suggest that oxidative stress, mitochondrial injury, lysosome membrane damage, allergic reactions, and vancomycin-associated tubular casts are all mechanistically involved [11,21]. Therefore, increased awareness of VIKI is still needed in clinical practice. In this context, several blood biomarkers have been proposed for predicting VIKI, but it is not known if they can precisely identify susceptible populations or detect VIKI at an earlier stage [8]. 

Despite the importance of urine biomarkers, accounting for urine dilution is important and may affect prognostication. For example, urine creatinine concentration reflects not only urine dilution but also muscle mass, which is itself independently associated with adverse clinical outcomes, and could influence the risk estimate [22]. In contrast, blood biomarkers are less likely to be affected by dilution and may prove important in the prognostication of VIKI. The ideal blood biomarker must be validated and functional to provide important information to gauge dosing and duration of treatment, able to discriminate VIKI versus other etiologies of AKI, able to assess the severity of VIKI, and able to assist the planning of therapy and management of AKI [23]. 

The objective of this systematic review and meta-analysis was to investigate the role of blood biomarkers and metabolomic profiling as diagnostic and prognostic predictors in pre-clinical studies of VIKI.

## 2. Material and Methods 

### 2.1. Protocol and Registration

The protocol was registered in the PROSPERO international prospective register of systematic reviews on 1 June 2022 (CRD42022337057). This systematic review and meta-analysis were reported according to the preferred reporting items for systematic reviews and meta-analyses (PRISMA) checklist (Supplemental Table S1) [24]. 

### 2.2. Inclusion and Exclusion Criteria

Animal studies were included. Urinary biomarker studies were excluded. Clinical studies, reviews, and non-English literature were excluded.

### 2.3. Outcomes of Interest

The primary outcome was to investigate the relationship between the burden of VIKI as measured by blood biomarkers and metabolomic profiling. Secondarily, the association of the dose of vancomycin with VIKI was investigated within the included studies.

### 2.4. Search Strategy

The search strategy was intended to explore all available published pre-clinical studies from January 2000 to May 2022, and was designed by four authors (AC, TM, NP, TX). A comprehensive initial search was employed in PubMed (MEDLINE) for articles containing any of the following terms in the abstract or title: the MeSH^®^ terms vancomycin, the wildcard terms vancomyc* or vanco*, or any of the following terms; renal, kidney, nephrotoxicity, creatinine, glomerular filtration rate, tubular, injury, animal, mice, rat, swine, biomarkers, marker, pharmacokinetic, toxicodynamic, cellular death. We then searched for those articles which meet the above criteria AND any of the following MeSH^®^ terms: preclinical model, translational, or human translation or the following non-MeSH terms: complications, outcome, or survival. Another search was conducted with the reference lists of all identified reports and articles for additional studies.

### 2.5. Data Extraction 

The data from each study were extracted by two independent authors (AC, TM) with a customized format. Any disagreements between the two independent authors were resolved by two other authors (NP, TX). Publication details (authors, year), study information (design, population, follow-up, inclusion-exclusion criteria, and number of cases/cohort-size, subgroups, dose), VIKI, and outcome were extracted in a pre-designed Excel spreadsheet. Authors of studies with missing data were contacted in an attempt to obtain relevant data. 

### 2.6. Assessment of Methodological Quality

Articles identified for retrieval were assessed by three independent authors (AC, TM, NP) for methodological quality before inclusion in the review using a standardized critical appraisal tool. Any disagreements between the authors appraising the articles were resolved through discussion with the other authors.

### 2.7. Data Analysis and Synthesis

The standardized mean differences (SMD) between animals belonging to control group and animals treated with vancomycin was used as the measure of effect size in the random-effects models. Between-study heterogeneity was assessed by The I^2^ score was used to establish the levels of between-study heterogeneity. Values over 50% were considered high. No estimation of possible publication bias or any subgroup analysis was undertaken due to the small number of available studies. R v. 4.1 (R foundation) was utilized for the included analyses. The threshold of statistical significance was set at *p*-values less than 0.05.

## 3. Results

**Figure 1:**
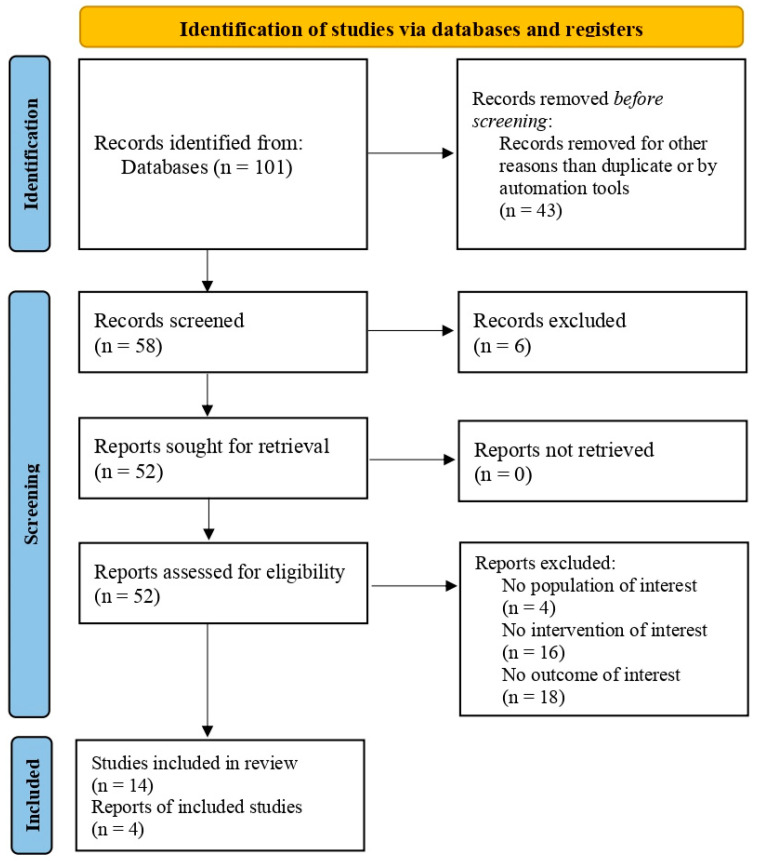
Preferred Reporting Items for Systematic Reviews and Meta-Analyses (PRISMA) diagram.

Altogether, 101 relevant citations were identified and screened. Of the 101 citations, 14 studies were selected for full review based on the abstract and included in our final assessment for possible data extraction (Figure 1, Table 1) [25,26,27,28,29,30,31,32,33,34,35,36,37,38]. In total, data extraction, used in data synthesis, was possible in four studies including a total of 58 animals [25,28,32,34]. 

### 3.1. Synthesis including All Data

There were four studies for the same species, dosage, duration of vancomycin administration and measurement only for serum creatine and blood urea nitrogen (BUN) in rats. Those rats were given 200 mg b.i.d. for 7 days [25,28,32,34]. 

**Figure 2:**
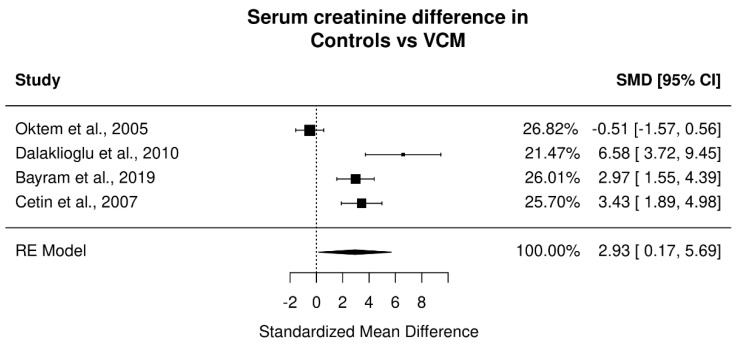
Effect of vancomycin on serum creatinine in animals receiving vancomycin vs. controls. Presented in plots Standardized Mean Difference and 95% confidence intervals [25,28,32,34].

**Figure 3:**
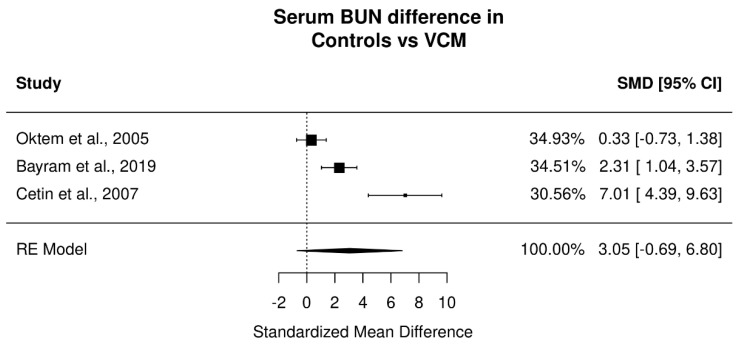
Effect of vancomycin on serum blood urea nitrogen in animals receiving vancomycin vs. controls. Presented in plots Standardized Mean Difference and 95% confidence intervals [25,32,34].

A statistically significant increase was found between serum creatinine in the vancomycin group compared to controls (*p* = 0.037; Standardized Mean Difference: 2.93; 95% CI: 0.17 to 5.69). Very high heterogeneity was present (I^2^ = 92.11%). Serum BUN levels were not significantly different between control and vancomycin groups (*p* = 0.11; SMD: 3.05; 95% CI: 0.69 to 6.8) with very high heterogeneity (I^2^ = 94.84%). Forest plots for the different measurements are presented in Figure 2 and Figure 3 and a summary of the results is presented in Table 2.

We did not identify experimental studies using metabolomic analyses in animals with VIKI.

### 3.2. Secondary Outcome

The data were insufficient for assessing the secondary outcome.

### 3.3. Risk of Bias, Quality of Evidence 

Assessment by the CAMARADES checklist for study quality is presented in Supplemental Table S2. In addition, all studies scored high risk of bias in the first eight items of SYRCLE’s risk of bias tool.

## 4. Discussion

Vancomycin-induced AKI significantly affects the course and outcome of critically ill patients, yet this systematic review and meta-analysis confirms that the available evidence from experimental studies investigating blood biomarkers cannot improve clinical practice. Several rodent studies were identified assessing a variety of biomarkers, but the synthesis of data on VIKI was meaningful only for creatinine and serum BUN. These findings highlight the underuse of other experimental models, e.g., swine, and the need for more pragmatic translational research. 

Vancomycin-induced AKI is a serious complication, especially among septic patients and in a dose-response relationship, leading to high rates of mortality and morbidity [38]. The exact mechanism of VIKI remains unclear, but oxidative stress and mitochondrial dysfunction have been observed in vancomycin treated animals and in cell culture studies [39]. At the same time, early detection of VIKI is critically important in order to prevent kidney injury and improve outcomes. In the present analysis, data analysis and synthesis were possible only for a few studies, revealing a significant increase in serum creatinine after exposure to vancomycin. UpToDate, the current KDIGO clinical practice guidelines for acute kidney injury recommend the use of serum creatinine as a biomarker. However, creatinine is slow to reach concentrations that are diagnostic for AKI; often kidney damage is not identified for several days. Notably, all studies that were included in synthesis of data used rodents and 200 mg kg^−1^ (an allometric equivalent of ~30 mg/kg/day in humans) of vancomycin for 7 days [25,28,32,34], demonstrating that the rodent model results in similar time to detection of kidney injury in humans by serum creatinine [40]. This slow detection of damage, when it is known that injury happens earlier [41], should be improved. 

Several studies so far have shown the importance of urinary biomarkers in VIKI, but their application in clinical practice remains difficult. A major cause for this may be the species that are usually used in VIKI studies, i.e., rats or mice. Undoubtedly, the choice of the animal model in translational research is not always a simple task, as there are multiple parameters that have to be considered when designing an experiment [42]. Although the rat and mouse have been linked to human outcomes, physiological characteristics differ significantly from those of humans. In contrast, the use of swine in biomedical research has increased in the last decades due to similarities in cardiac anatomy and physiology with humans [42]. Additionally, swine offer the ability to study human durations of treatment in scenarios in which human patients are most vulnerable (i.e., critically ill and sedated). As biomarker and metabolomic profiles identified from blood are highly preferable [43], swine offer greater physiologic similarity to humans and allow serial biopsy and moderate volume phlebotomy in a model that most closely mimics the disease in humans (i.e., injury in patients that are critically ill) [44,45,46]. 

The field of metabolomics also holds significant potential for precision medicine and earlier identification of disease states. Recent studies have investigated the metabolomics related to AKI, in the hope of earlier and more specific identification of kidney injury [47,48,49]. Early identification of AKI in patients receiving vancomycin could enable clinicians to stop or change therapy before irreversible toxicity results. In a study exploring the potential mechanism of VIKI in rats, the authors performed urinary metabolomic analysis on animals treated with intraperitoneal vancomycin for 2, 4, 7 days, and control (saline) [50]. They reported that amino acid metabolism and energy metabolism were disturbed in rats with vancomycin-associated nephrotoxicity. However, there was not a control group of a different nephrotoxic agent and thus, that study discuss AKI rather VIKI markers (whether urinary or serum). Our group recently showed that kidney function decline was observed among rats that received vancomycin only but not those that received piperacillin-tazobactam or vancomycin plus piperacillin-tazobactam [35]. We also shown that polymyxin B causes increased amounts of urinary taurine on day 1, which then normalizes to baseline concentrations [43]. Of note, the taurine response correlated with KIM-1, a proximal tubule kidney injury biomarker, indicating that taurine could be involved as a physiologic protective response to the insult by polymyxin B. In the present study, data synthesis was possible only for serum creatinine and BUN. As novel blood biomarkers have not been explored and VIKI is the result of drug accumulation in proximal tubule cells, triggering cellular oxidative stress and apoptosis [11], taurine could be also a promising metabolomic marker of VIKI.

Nanomedicine can be another promising tool in diagnosis and treatment of VIKI in swine. Nanoparticles are an attractive candidate due to their ability to act as theranostic tools that can carry drugs and enhance image contrast, while nanoparticle-based point-of-care systems can provide novel physiological information and control over the rate and location of drug release [51]. The application of naturally occurring nanostructures such as exosomes and cell-engineered nanovesicles can help identify and regenerate damaged tissues after vancomycin administration. The combined use of nanobiomaterials and metabolomics could optimize management of VIKI, while the application of microfluidic devices mimicking the nephrons and other kidney cells could act as an artificial substitute for studying VIKI [52]. However, the development of a scaffold which exactly mimics the natural extracellular matrix is still a matter of question. 

Another critical player in in AKI is mitochondria, which have dual roles as the primary source of energy for each cell and as a key regulator of cell death. Research has shown that their structure can change due to ischemia-induced ATP depletion and membrane potential reduction [53]. In addition, direct and indirect evidence suggest that the opening of mitochondrial permeability transition pores (mPTP) is a key event that contributes to organ progression, including the kidneys, through releasing pro-apoptotic mediators, such as cytochrome c [54,55,56]. Furthermore, the protein Drp1 is rapidly activated while the proteins Mfn and Opa1 are decreased following AKI, resulting in mitochondrial fragmentation [57,58]. A dysregulation of mitochondrial biogenesis is also observed in AKI that alters gene expression, e.g., PGC-1α or NRF1/2 [58,59,60]. VIKI-induced mitochondrial injury may release ROS, DNA, and cardiolipin, which can activate NOD-like receptors and elevate the levels of proinflammatory cytokines and chemokines that may, in turn, induce and/or aggravate renal fibrosis and tubular damage [53]. A new swine model could enhance research on the role of mitochondria related markers in VIKI.

## 5. Limitations

This analysis investigated the role of biomarkers and metabolomic profiling as diagnostic and prognostic predictors in pre-clinical studies of VIKI. Consequently, it included studies investigating a highly heterogeneous group of animals including different doses and regimens. Due to the lack of data, the synthesis of all the available knowledge on the specific outcomes was difficult. In addition, as it is stated in the data analysis section, no statistical assessment of publication bias could be performed due to the limited number of studies. For the same reason, a subgroup analysis was inappropriate. 

## 6. Conclusions

The available evidence on blood biomarkers coming from experimental studies cannot improve clinical practice and early diagnosis of VIKI. Translational research on VIKI should be advanced to a highly relevant critically ill swine model which can provide high-quality assessment of blood biomarkers including metabolomic analyses.

## Figures and Tables

**Table 1:** Major blood biomarkers in animal studies of vancomycin-induced acute kidney injury.

Author Year	Species	VCM				Biomarker (Units)	Control	VCM Group
		Administration	Dose (mg kg^−1^)	Frequency	Duration			
Oktem 2005	Rats	Intraperitoneally	200	b.i.d.	7 days	Creatinine (mg dL^−1^)	0.44 ± 0.06	0.41 ± 0,05
						BUN (mg dL^−1^)	18.8 ± 8.21	21.2 ± 5.1
Naghibi 2007	Rats	Intraperitoneally	200	b.i.d.	7 days	Creatinine (mg dL^−1^)	×2.5	NA
						BUN (mg dL^−1^)	×5	NA
Dalaklioglu 2010	Rats	Intraperitoneally	200	b.i.d.	7 days	Creatinine (mg dL^−1^)	0.8 ± 0.04	3.38 ± 0.51
						BUN (mg dL^−1^)	8.07 ± 0.75	53.87 ± 10.11
Takigawa 2017	Mice	Intraperitoneally	400	q.d.	3 days	Creatinine (mg dL^−1^)	×5.4	NA
						BUN (mg dL^−1^)	×4.6	NA
	Mice	Intraperitoneally	400	q.d.	5 days	Creatinine (mg dL^−1^)	NS	NA
						BUN (mg dL^−1^)	×2.5	NA
	Mice	Intraperitoneally	400	q.d.	7 days	Creatinine (mg dL^−1^)	×4.0	NA
						BUN (mg dL^−1^)	×3.3	NA
Qu 2018	Rats	Intraperitoneally	200	b.i.d.	7 days	Creatinine (mg dL^−1^)	N/A	NA
						BUN (mg dL^−1^)	N/A	NA
Bayram 2019	Rats	Intraperitoneally	200	b.i.d.	7 days	Creatinine median (IQR) (mg dL^−1^)	0.33 (0.29–0.34)	0.52 (0.38–0.54)
						BUN (mg dL^−1^)	21.2 ± 3.5	35.18 ± 7.3
El Bohi 2021	Rats	Intraperitoneally	443.6	q.o.d.	14 days	Creatinine (mg dL^−1^)	0.83 ± 0.03	1.28 ± 0.1
						BUN (mg dL^−1^)	27.75 ± 1.55	74.25 ± 2.14
						Uric acid (mg dL^−1^)	6.16 ± 0.13	9.54 ± 0.23
Cetin 2007	Rats	Intraperitoneally	200	b.i.d.	7 days	Creatinine (mg dL^−1^)	0.43 ± 0.09	0.97 ± 0.19
						BUN (mg dL^−1^)	16.25 ± 2.38	41.25 ± 4.13
Bruniera 2014	Rats	Intravenously	10 (5 mg ml^−1^)	q.d.	3 days	Creatinine (mg dL^−1^)	0.5 ± 0.1	0.4 ± 0.1
						BUN (mg dL^−1^)	49.0 ± 6.9	46.1 ± 6.0
			10 (10 mg ml^−1^)	q.d.	3 days	Creatinine (mg dL^−1^)	0.5 ± 0.1	0.5 ± 0.1
						BUN (mg dL^−1^)	49.0 ± 6.9	50.9 ± 9.0
			10 (5 mg ml-^1^)	q.d.	7 days	Creatinine (mg dL^−1^)	0.6 ± 0.1	0.5 ± 0.1
						BUN (mg dL^−1^)	50.9 ± 9.1	50.0 ± 3.8
			10 (10 mg ml^−1^)	q.d.	7 days	Creatinine (mg dL^−1^)	0.6 ± 0.1	0.6 ± 0.1
						BUN (mg dL^−1^)	50.9 ± 9.1	44.0 ± 4.8
Shi 2021	Mice	Intraperitoneally	400	q.d.	3 days ^#^	Creatinine (mg dL^−1^)	69.2%	NA
						BUN (mg dL^−1^)	110%	NA
O’Donnell 2018	Rat	Intraperitoneally	150	b.i.d.	14 days	Creatinine (mg dL^−1^)	365.6 ± 67.3	237.4 ± 23.7
						BUN (mg dL^−1^)	26.8 ± 16.4	32.7 ± 3.5

^#^ Samples were collected at the 8th day.

**Table 2:** Summary of meta-analysis results.

Parameter	Number of Studies	N (Total)	Estimate (SMD)	*p*-Value	95% CI	I^2^	Q	*p* (Q)
Serum creatinine	4	58	2.93	0.037	0.17 to 5.69	92.11%	35.6	<0.001
Serum BUN	3	46	3.05	0.11	−0.69 to 6.80	94.84%	23.16	<0.001

## Data Availability

Data can be made available upon request after publication through a collaborative process. Researchers should provide a methodically sound proposal with specific objectives in an approval proposal. Please contact the corresponding author for additional information.

## Supplementary Materials

The following supporting information can be downloaded at: https://www.mdpi.com/article/10.3390/jpm12091397/s1, Table S1: PRISMA Checklist; Table S2: CAMARADES checklist of included studies.
