# Supplementary material for: Blood Biomarkers and Metabolomic Profiling for the Early Diagnosis of Vancomycin-Associated Acute Kidney Injury: A Systematic Review and Meta-Analysis of Experimental Studies
Source: J Pers Med. 2022 Aug 28;12(9):1397. doi: 10.3390/jpm12091397 (PMC9505572; doi:10.3390/jpm12091397)
Supplement: Supplementary file 1 [file jpm-12-01397-s001.zip › jpm-1879208-supplementary/Supplemental Table S2.pdf]

Supplemental Table S2. CAMARADES checklist of included studies

**CAMARADES CHECKLIST**	**Oktem et al. (2005)**	**Cetim et al. (2007)**	**Dalakioglu et al. (2010)**	**Bayram et al. (2019)**
Publication in peer-reviewed journal	Y	Y	Y	Y
Statement of control of temperature	Y	Y	N	Y
Randomization of treatment or control	Y	Y	Y	N
Allocation concealment	N	N	N	N
Blinded assessment of outcome	N	N	N	N
Avoidance of anesthetics with marked intrinsic properties	Y	Y	N	N
Use of animals with hypertension or diabetes	N	N	N	N
Sample size calculation	N	N	N	N
Statement of compliance with regulatory requirements	Y	Y	Y	Y
Statement regarding possible conflict of interest	N	N	N	N
Total (on 10)	5	5	3	3
